# Optimized ventilation strategy for surgery on patients with obesity from the perspective of lung protection: A network meta-analysis

**DOI:** 10.3389/fimmu.2022.1032783

**Published:** 2022-10-18

**Authors:** Jing Wang, Jie Zeng, Chao Zhang, Wenwen Zheng, Xilu Huang, Nan Zhao, Guangyou Duan, Cong Yu

**Affiliations:** ^1^ Department of Anesthesiology, The Stomatology Hospital Affiliated Chongqing Medical University, Chongqing, China; ^2^ Chongqing Key Laboratory of Oral Diseases and Biomedical Sciences, Chongqing, China; ^3^ Chongqing Municipal Key Laboratory of Oral Biomedical Engineering of Higher Education, Chongqing, China; ^4^ Department of Anesthesiology, The Second Affiliated Hospital, Chongqing Medical University, Chongqing, China

**Keywords:** immune response, inflammation, intraoperative ventilation strategy, obesity, pulmonary atelectasis

## Abstract

**Objectives:**

New ventilation modes have been proposed to support the perioperative treatment of patients with obesity, but there is a lack of consensus regarding the optimal strategy. Therefore, a network meta-analysis update of 13 ventilation strategies was conducted to determine the optimal mode of mechanical ventilation as a protective ventilation strategy decreases pulmonary atelectasis caused by inflammation.

**Methods:**

The following databases were searched: MEDLINE; Cochrane Library; Embase; CINAHL; Google Scholar; and Web of Science for randomized controlled trials of mechanical ventilation in patients with obesity published up to May 1, 2022.

**Results:**

Volume-controlled ventilation with individualized positive end-expiratory pressure and a recruitment maneuver (VCV+PEEPind+RM) was found to be the most effective strategy for improving ratio of the arterial O_2_ partial pressure to the inspiratory O_2_ concentration (PaO_2_/FiO_2_), and superior to pressure-controlled ventilation (PCV), volume-controlled ventilation (VCV), volume-controlled ventilation with recruitment maneuver (VCV+RM), volume-controlled ventilation with low positive end-expiratory pressure (VCV+lowPEEP), volume-controlled ventilation with lower positive expiratory end pressure (PEEP) and recruitment maneuver (VCV+lowPEEP+RM), and the mean difference [MD], the 95% confidence intervals [CIs] and [quality of evidence] were: 162.19 [32.94, 291.45] [very low]; 180.74 [59.22, 302.27] [low]; 171.07 [40.60, 301.54] [very low]; 135.14 [36.10, 234.18] [low]; and 139.21 [27.08, 251.34] [very low]. Surface under the cumulative ranking curve (SUCRA) value showed VCV+PEEPind+RM was the best strategy for improving PaO_2_/FiO_2_ (SUCRA: 0.963). VCV with high positive PEEP and recruitment maneuver (VCV+highPEEP+RM) was more effective in decreasing postoperative pulmonary atelectasis than the VCV+lowPEEP+RM strategy. It was found that volume-controlled ventilation with high positive expiratory end pressure (VCV+highPEEP), risk ratio [RR] [95% CIs] and [quality of evidence], 0.56 [0.38, 0.81] [moderate], 0.56 [0.34, 0.92] [moderate]. SUCRA value ranked VCV+highPEEP+RM the best strategy for improving postoperative pulmonary atelectasis intervention (SUCRA: 0.933). It should be noted that the quality of evidence was in all cases very low or only moderate.

**Conclusions:**

This research suggests that VCV+PEEPind+RM is the optimal ventilation strategy for patients with obesity and is more effective in increasing PaO_2_/FiO_2_, improving lung compliance, and among the five ventilation strategies for postoperative atelectasis, VCV+highPEEP+RM had the greatest potential to reduce atelectasis caused by inflammation.

**Systematic Review Registration:**

https://www.crd.york.ac.uk/PROSPERO/, identifier CRD42021288941.

## 1. Introduction

The steady increase in obesity in adults is producing clinical conditions that are prevalent worldwide (1). As the number of individuals with obesity increases, so does the number of patients with obesity undergoing surgery and requiring mechanical ventilation. Even if lung function is normal, patients under general anesthesia are prone to complications such as impaired respiratory gas exchange and mechanics or pulmonary atelectasis. Pulmonary atelectasis is a common complication after patients with obesity have been mechanically ventilated. It not only reduced the oxygenation of blood and lung compliance but also caused local tissue inflammation, immune dysfunction and injury to the alveolar-capillary barrier, leading to reduced lung fluid clearance and increased lung injury (2). This condition is associated with the local synthesis and secretion of cytokines that stimulate inflammatory responses. Local immune dysfunction in atelectasis mainly involves cytokine and inflammatory responses. In addition, atelectasis alone can stimulate alterations in the immune functions of key cells (3). It enhanced alveolar macrophage cytokine secretion in rats (4), impaired phagocytosis of bacteria by macrophages in piglets *in vitro* (5), and reduced local lymphocytes bronchoalveolar functions in the dog (6). Different immune transcriptome patterns were recorded in atelectasis vs. sheep lungs that were ventilated, with fewer NF-κB-related genes being involved in atelectasis (7). Thus, the finding of similarities in inflammatory injury in atelectasis and ventilated lungs may stem from different responses to various cytokines (8). Patients with obesity have accumulations of fat that can limit chest wall compliance and decrease total lung capacity. This can reduce lung compliance and functional residual capacity, lead to inadequate O_2_ storage and impaired respiratory mechanics during ventilation, thus contributing to postoperative pulmonary atelectasis (9), requiring longer hospital stays for these patients (10). Therefore, it is essential to select the optimal ventilation strategy for patients with obesity to improve intraoperative oxygenation and reduce postoperative pulmonary atelectasis.

In a previous meta-analysis of ventilation strategies, it was concluded that volume-controlled ventilation with high positive expiratory end pressure and recruitment maneuvers (VCV+highPEEP+RM) were better than other strategies with regard to improvements in the ratio of the arterial O_2_ partial pressure to the inspiratory O_2_ concentration (PaO_2_/FiO_2_), intraoperative lung compliance, and in the prevention atelectasis during anesthesia inpatients with obesity. In contrast, pressure-controlled ventilation with lower positive expiratory end pressure (PCV+lowPEEP) was least able to improve oxygenation for patients with obesity (11). However, no comprehensive comparison of pulmonary atelectasis and lung compliance have been conducted. A recent multicenter, large-sample study reported that in patients with obesity undergoing general anesthesia, VCV+highPEEP+RM did not reduce postoperative pulmonary atelectasis compared to volume-controlled ventilation with low positive expiratory end pressure (VCV+lowPEEP) (12). In addition, new ventilation strategies have emerged in the last five years that have not yet been evaluated in this way. Excessively high positive expiratory end pressure (PEEP), however, can elicit barotrauma and hemodynamic instability. Therefore, the lowest value of PEEP that maintains the alveoli open has been termed “ideal PEEP”. Individualized PEEP involved determining optimal PEEP according to patients specific characteristics, including lung dynamic compliance and the driving pressure (13). Based on the above findings, we provide an updated meta-analysis of ideal strategies for the mechanical ventilation of patients with obesity.

## 2. Materials and methods

The current network meta-analysis follows the 2020 PRISMA guidelines (14) and is registered with PROSPERO (CRD42021288941).

### 2.1 Literature search

JW and JZ (2 authors) each searched MEDLINE, Cochrane Library, Embase, CINAHL, Google Scholar and Web of Science databases independently to identify appropriate articles published from the start until May 1, 2022. There were no language restrictions. Keywords in the PubMed data repository were searched as follows: (ventilation OR respiration OR pulmonary gas exchange) OR (tidal volume) OR (positive end-expiratory pressure OR positive end-expiratory positive pressure OR PEEP) OR (recruitment-action) AND (obese OR obesity OR bariatric OR overweight OR overnutrition) AND (surgery OR surgical OR operation OR operative). We also reviewed the reference lists of previously published reviews and meta-analyses to properly screen for further relevant studies.

### 2.2 Inclusion and exclusion criteria

JW and JZ independently assessed the eligibility for a study to be included from the article title, abstract and full text. A third author (CY) was invited to mediate a decision when there was disagreement. Inclusion criteria were (1): An intervention was defined as intraoperative ventilation strategy that was based on a low VT (≤ 8 mL/kg) and the predicted body weight (PBW); articles were allocated to groupings according to the PEEP level (low [≤ 5 cmH_2_O], high [≥ 10 cmH_2_O]) and with or without recruitment maneuvers (RMs); (2) a body mass index (BMI) ≥ 30 kg/m^2^ and adults aged between 18 and 65 years old; (3) randomized controlled trials (RCTs); and a requirement for intraoperative mechanical ventilation. Exclusion criteria were: non-invasive ventilation; BMI values < 30 kg/m^2^; pediatric trials; reviews; observational studies; case reports of retrospective studies; animal studies; and repeat studies.

### 2.3 Outcome measurements and data extraction

Primary outcomes were the intraoperative PaO_2_/FiO_2_ ratio and postoperative pulmonary atelectasis. Secondary outcomes included intraoperative lung compliance. If multiple measurements of the same outcome indicator occurred during the operation, the last measured value was taken. When necessary, data extraction forms were sent to the original authors to request complete data or missing data. If no author response, the mean was deemed to equivalent to the median and the SD was appropriately estimated.

### 2.4 Evaluation of article quality

Two authors (CZ and WWZ) each determined the risk of bias using the Cochrane risk-of-bias tool for randomized trials (RoB2); a third author (CY) was invited to resolve any disagreements. The Cochrane risk-of-bias tool evaluates 7 parameters: selection (including random sequence generation and allocation concealment), implementation (including blinding of investigators and subjects), measurement (blinded assessment of study outcomes), follow-up (completeness of outcome data), reporting (selective study reporting of results), and other (other sources of bias); each of these items was classified as “low risk of bias”, “unclear” or a “high risk of bias” (15).

Two authors (XLH and GYD) independently evaluated the credibility of the network meta-analysis using the Confidence in Network Meta Analysis (CINeMA) web application, which consists of 6 parameters: within-study and across-study bias, indirectness, imprecision, heterogeneity, and inconsistency. Each parameter was assessed to be of “no”, “minor”, or “major” for concern and an assessment of confidence with each outcome (high, medium, low, or very low) (16). When there was disagreement between the two evaluators, a third author (CY) was invited to discuss and contribute to the final decision.

### 2.5 Statistical analysis

Bayesian network meta-analysis was used for each outcome to compare the effects of the various ventilation strategies. STATA/MP software (ver. 16) was employed to generate network meta-plots and analyze the results. In each network diagram, line thickness was proportional to the number of trials used for comparison, and the node size corresponded to the total sample size. The effects of each mechanical ventilation strategy were measured according to the intraoperative PaO_2_/FiO_2_ and lung compliance in patients with obesity using the mean difference (MD) and the 95% CIs as parameters.

The risk ratio (RR) and 95% CIs were used to evaluate the incidence of postoperative pulmonary atelectasis. This analysis used the surface under the cumulative ranking curve (SUCRA) to rank the effects of different ventilation strategies on outcome indicators. Larger SUCRA values indicate a greater effect of the ventilation modality on outcome indicators and a superior clinical choice. A SUCRA score of 100% indicates that the intervention was effective, and a score of 0% indicates that the intervention was ineffective (17). Publication bias was evaluated using funnel plots, where points that were evenly distributed indicated a small bias for the included RCTs. A design-by-treatment model was employed to determine global inconsistency across the network meta-analysis and the node splitting method to evaluate a local inconsistency in performing the consistency analysis. The results are given as *P*-values, inconsistency factors and 95% CIs. If the *P*-value was > 0.05 and the inconsistency factor was near to 0, the direct comparison evidence was deemed to be consistent with that of the indirect comparison (18).

### 2.6 Heterogeneity analysis

Systematic heterogeneity was assessed by testing *I^2^* values and ratios with 95% CIs. *I^2^* equal to 0% a represented no heterogeneity, *I^2^* equal to 25% indicated low heterogeneity, *I^2^* equal to 50% represented moderate heterogeneity, and *I^2^* equal to 75% high heterogeneity. *I^2^* values greater than 50% are considered the cut-off point for determining the presence of considerable heterogeneity (19).

### 2.7 Sensitivity analysis

Initially 23 RCTs were included that examined a total of 13 types of ventilation. Sensitivity analysis was conducted using the one-by-one elimination method.

## 3. Results

**Figure 1:**
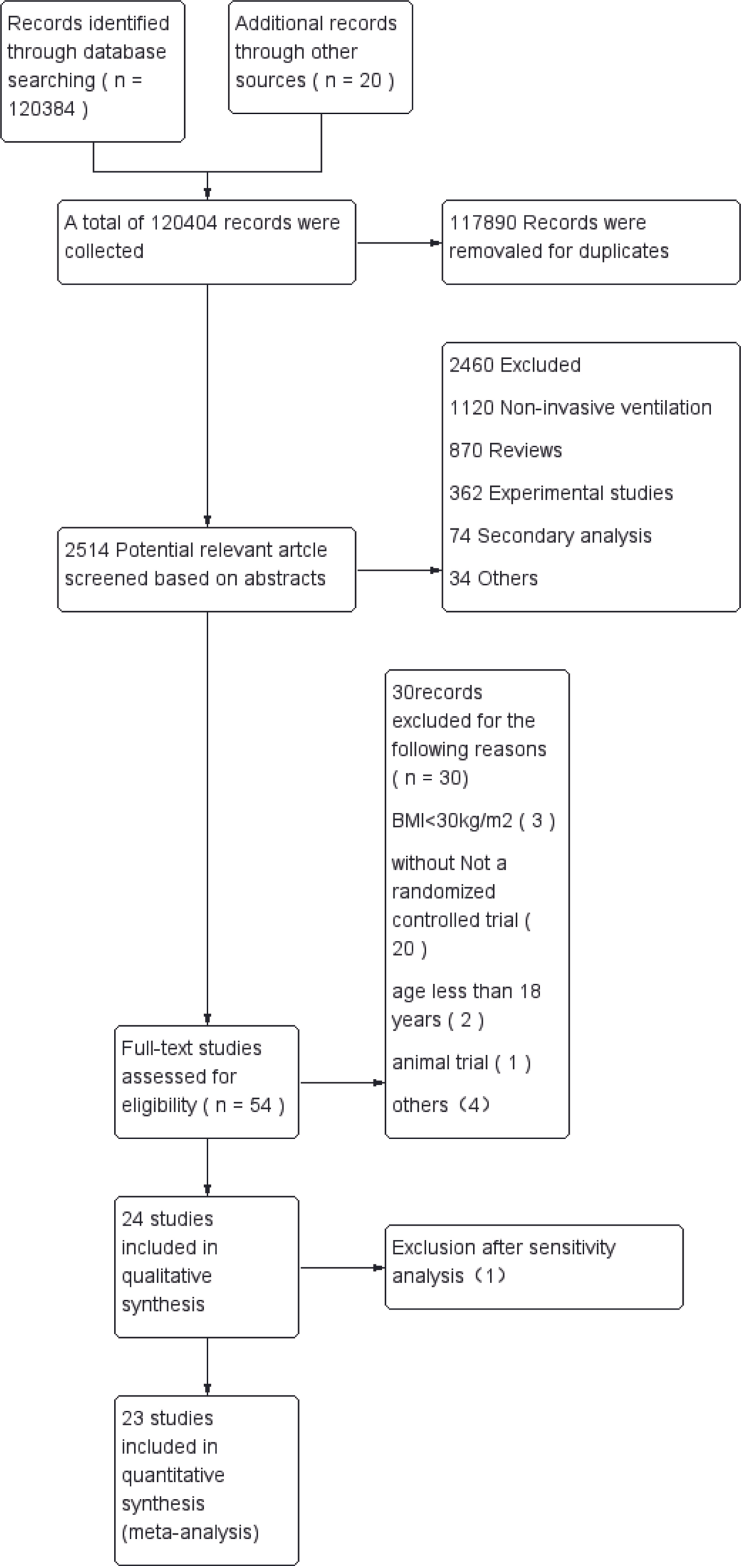
Flow chart of literature search.

A total of 120,404 relevant articles were found by searching the databases and web pages. After excluding duplicate literature, 2,514 articles remained. After an initial screen of titles and abstracts, 54 potentially eligible studies were identified for which the full text was retrieved and a more detailed evaluation made. After the full-text assessment, 30 studies were found that did not meet the inclusion criteria. We also excluded 1 article after sensitivity analysis. Finally, 23 RCTs were included (12, 20–41), which involved a total of 3,364patients with obesity who were randomized to 13 ventilation strategies (**Figure 1**). Information extracted from each article included the first author, publication year, design of the study, surgery type, patient data (age, country, American Society of Anesthesiologists physical status classification [ASA], BMI and sample size), ventilation strategy, outcome measures (PaO2/FiO2, intraoperative lung compliance, postoperative pulmonary atelectasis) (**Table 1**).

**Table 1:** Basic characteristics of the included studies.

Study (year)	Country	Type of surgery	Ventilation strategies	Patients (n)	Age(years)	ASA	Outcome	Reference
Baerdemaeker (2008)	Belgium	Laparoscopic surgery	VCV+lowPEEP (5 cmH_2_O)	12	35.1 ± 10.4	I-II	PIO_2_/FiO_2_, compliance	(20)
			PCV+lowPEEP (5 cmH_2_O)	12	41.7 ± 7.6	I-II		
Chalhoub (2006)	Beirut	Open bariatric surgery	VCV+lowPEEP (8 cmH_2_O)	26	36.0 ± 11.3	/	PIO_2_/FiO_2_	(21)
			VCV+lowPEEP+RM (8 cmH_2_O)	26	36.2 ± 10.2			
Sprung (2009)	America	Open bariatric surgery	VCV+lowPEEP (4 cmH_2_O)	9	48 ± 9	/	PIO_2_/FiO_2_	(22)
			VCV+highPEEP+RM (12 cmH_2_O)	8	52 ± 9			
Reinius (2009)	Sweden	Open gastric bypass surgery	VCV+RM	10	37 ± 10		PIO_2_/FiO_2_, pulmonary atelectasis, compliance	(23)
			VCV+highPEEP (10 cmH_2_O)	10	40 ± 10	II-III		
			VCV+highPEEP+RM (10 cmH_2_O)	10	35 ± 8			
Cadi (2008)	France	Laparoscopic obesity surgery	VCV+lowPEEP (5 cmH_2_O)	18	40 ± 12	/	PIO_2_/FiO_2_, compliance	(24)
			PCV+lowPEEP (5 cmH_2_O)	18	40 ± 9			
Whalen (2006)	America	Laparoscopic bariatric surgery	VCV+lowPEEP (4 cmH_2_O)	10	38 ± 11	/	PIO_2_/FiO_2_, pulmonary atelectasis	(25)
			VCV+lowPEEP+RM (10 cmH_2_O)	10	44 ± 9			
Tafer (2008)	France	Laparoscopic bariatric surgery	VCV+highPEEP (10 cmH_2_O)	13	39 ± 8	I-III	Compliance	(26)
			VCV+highPEEP+RM (10cmH_2_O)	13	38 ± 10			
Talab (2009)	Arabia	Laparoscopic bariatric surgery	VCV+RM	22	29.3 ± 9.2	/	Pulmonary atelectasis	(27)
			VCV+highPEEP (10 cmH_2_O)	22	28.9 ± 9.3			
			VCV+lowPEEP+RM (5 cmH_2_O)	22	28.9 ± 8.5			
Defresne (2014)	Belgium	Open gastric bypass surgery	VCV+highPEEP (10 cmH_2_O)	_2_25	41.3 ± 3.8	II-III	Compliance	(28)
			VCV+highPEEP+RM (10 cmH_2_O)	25	41.2 ± 2.5			
Toker (2019)	Arabia	Open gastric bypass surgery	VCV+lowPEEP (5 mmHg)	50	51 ± 7.7	II-III	Compliance	(29)
			PCV-CG+lowPEEP (5 mmHg)	50	50.5 ± 9.4			
Bluth (2019)	Germany	Open/Laparoscopic	VCV+lowPEEP (4 mmHg)	987	48.9 ± 13.3	I-II	Pulmonary atelectasis	(12)
			VCV+highPEEP+RM (12 mmHg)	989	48.9 ± 13.8			
Nestler (2017)	Germany	Laparoscopic surgery	VCV+lowPEEP (5 cmH_2_O)	25	44.8 ± 11.2	I-III	Compliance	(30)
			VCV+PEEPind+RM	25	44.3 ± 10.2			
Ghodraty (2021)	America	Laparoscopic surgery	VCV+lowPEEP (5 cmH_2_O)	30	38.5 ± 8.8	I-II	PIO_2_/FiO_2_	(31)
			PCV	30	35.7 ± 9.8			
Wei (2018)	China	Laparoscopic surgery	VCV	12	37.4 ± 11.6	II-III	PIO_2_/FiO_2_, compliance	(32)
			VCV+RM	11	33.8 ± 8.8			
			VCV+lowPEEP+RM (8 cmH_2_O)	11	37.6 ± 9.1			
Xu (2019)	China	Laparoscopic surgery	VCV	30	39.3 ± 11.7	II	PIO_2_/FiO_2_, compliance	(33)
			PCIRV	30	38.5 ± 13.4			
Van Hecke (2019)	Belgium	Laparoscopic bariatric surgery	VCV+highPEEP+RM (10cmH2O)	50	39.5 ± 4.5	II-III	PIO_2_/FiO_2_, compliance	(34)
			VCV+PEEPind+RM	50	41.6 ± 3.8			
Amaru (2021)	France	Cardiothoracic surgery	VCV+lowPEEP (5 cmH_2_O)	65	63 ± 10	/	PIO_2_/FiO_2_, pulmonary atelectasis	(35)
			VCV+lowPEEP+RM (5 cmH_2_O)	66	65 ± 10			
			VCV+highPEEP+RM (10 cmH_2_O)	61	63 ± 13			
Ozyurt (2019)	Turkey	Laparoscopic bariatric surgery	VCV	31	38.9 ± 10.7	/	PIO_2_/FiO_2_, compliance	(36)
			PCV	31	42.19 ± 9.6			
Zhao (2014)	China		VCV	10	48.4 ± 8.6		PIO_2_/FiO_2_	(37)
		Laparoscopic bariatric surgery	VCV+lowPEEP (5 cmH_2_O)	10	51.2 ± 8.1	I-II		
			VCV+highPEEP (10 cmH_2_O)	10	50.4 ± 6.7			
Hans (2007)	Belgium	Laparotomy/Laparoscopic	VCV	20	41.2 ± 11.2	/	PIO_2_/FiO_2_	(38)
			PCV	20	41.2 ± 11.2			
Tuncali (2018)	Turkey	Laparoscopic sleeve gastrectomy	VCV+highPEEP+RM (10 cmH_2_O)	55	33.8 ± 13.2	> III	Compliance	(42)
			VC-ERV+highPEEP+RM (10 cmH_2_O)	56	40.1 ± 12.7			
Zoremba (2010)	Germany	Elective minor peripheral surgery	PCV+lowPEEP (8 cmH_2_O)	34	46 ± 13	> II	PIO_2_/FiO_2_	(39)
			PSV+lowPEEP (8 cmH_2_O)	34	44 ± 12			
Simon (2021)	Germany	Laparoscopic bariatric surgery	VCV+lowPEEP (4 cmH_2_O)	44	46.5 ± 14.1		PIO_2_/FiO_2_, compliance	(41)
			VCV+highPEEP+RM (12 cmH_2_O)	21	43.6 ± 11.3	/		
			VCV+PEEPind+RM	25	44.9 ± 10.3			
Ali Said (2019)	India	Laparoscopic cholecystectomy	VCV	35	37.9 ± 9.7	II	PIO_2_/FiO_2_	(40)
			PCV	35	37.8 ± 9.0			

### 3.1 Primary outcomes

#### 3.1.1 Intraoperative PaO_2_/FiO_2_ ratio

**Figure 2:**
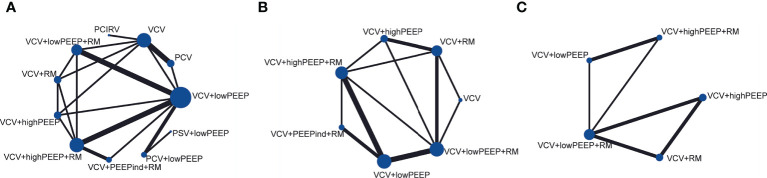
Network plots of main indicators. **(A)** PaO_2_/FiO_2_, **(B)** pulmonary atelectasis, **(C)** lung compliance.

**Figure 3:**
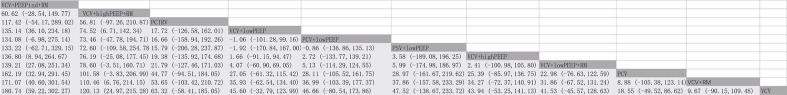
League table of PaO_2_/FiO_2_ under different ventilation strategies.

There were 17 RCTs (20–25, 31–41) involving a total of 985 patients with obesity, which assessed the MD of the overall effect size of the intraoperative PaO_2_/FiO_2_ across the 11 ventilation strategies (**Figure 2**). The network meta-analysis showed that the ventilation strategy volume-controlled ventilation with individualized positive end-expiratory pressure and recruitment maneuver (VCV+PEEPind+RM) was more efficacious in improving PaO_2_/FiO_2_ than pressure-controlled ventilation (PCV), volume-controlled ventilation (VCV), volume-controlled ventilation with recruitment maneuver (VCV+RM), VCV+lowPEEP or volume-controlled ventilation with lower positive expiratory end pressure and recruitment maneuver (VCV+lowPEEP+RM), MD [95% CI] and [quality of evidence]: 162.19 [32.94, 291.45] [very low]; 180.74 [59.22, 302.27] [low]; 171.07 [40.60, 301.54] [very low]; 135.14 [36.10, 234.18] [low]; and 139.21 [27.08, 251.34] [very low] (**Figure 3**). The SUCRA value ranked VCV+PEEPind+RM to be the best improved PaO_2_/FiO_2_ intervention (SUCRA: 0.963). The ventilation strategy VCV+highPEEP+RM has more potential to improve intraoperative PaO_2_/FiO_2_. The supporting information shows network plots of PaO_2_/FIO_2_, MD values and 95% CIs, credibility (**Figures S1, S4**) and SUCRA rankings for various ventilation strategies (**Figure 6**).

#### 3.1.2 Postoperative pulmonary atelectasis

**Figure 4:**
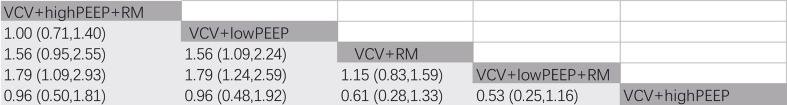
League table of pulmonary complications under different ventilation strategies.

In 4 RCTs (12, 23, 27, 35), a total of 2,264 patients with obesity were reported to have postoperative complications, and 5 ventilation strategies were examined (**Figure 2**). CT imaging of the chest was carried out on patient admission and also after discharge from the Post-anesthesia Care Unit. CT images were assessed for evidence of atelectasis, and classified into four main types depending on the thickness thus (1): lamellar atelectasis (< 3 mm); (2) plate atelectasis (3–10 mm); (3) segmental atelectasis (> 10 mm but less than one lobe); and (4) lobar atelectasis (atelectasis involving the entire lower lobe) (43). The network meta-analysis revealed that the ventilation strategy VCV+highPEEP+RM was more effective in reducing postoperative pulmonary atelectasis compared with the ventilation strategies VCV+lowPEEP+RM ([RR 0.56] 95% CI [0.39, 0.81] [moderate]). Compared with the ventilation strategy VCV+lowPEEP, the strategy VCV+lowPEEP+RM ([RR 0.56] 95% CI [0.34, 0.92] [moderate]) was more effective in reducing postoperative pulmonary atelectasis (**Figure 4**). SUCRA analysis showed that VCV+highPEEP+RM had the highest cumulative ranking (SUCRA 0.933). Network plots of pulmonary complication values, the ORs and 95% CIs, credibility (**Figures S2, S5**) and SUCRA rankings for various ventilation strategies are respectively shown in the supporting information (**Figure 6**).

### 3.2 Secondary outcomes

In 11 randomized trials (21, 23, 24, 26, 28–31, 33, 34, 36), a total of 630 patients with obesity were reported to have lung compliance values across 10 ventilation strategies (**Figure 2**). The quasistatic compliance of the respiratory system was evaluated as:

Tidal volume/inspiratory plateau pressure - end-expiratory pressure during no-flow at end-inspiration and end-expiration.

**Figure 5:**
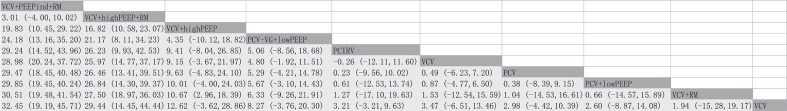
League table of pulmonary compliance under different ventilation strategies.

**Figure 6:**
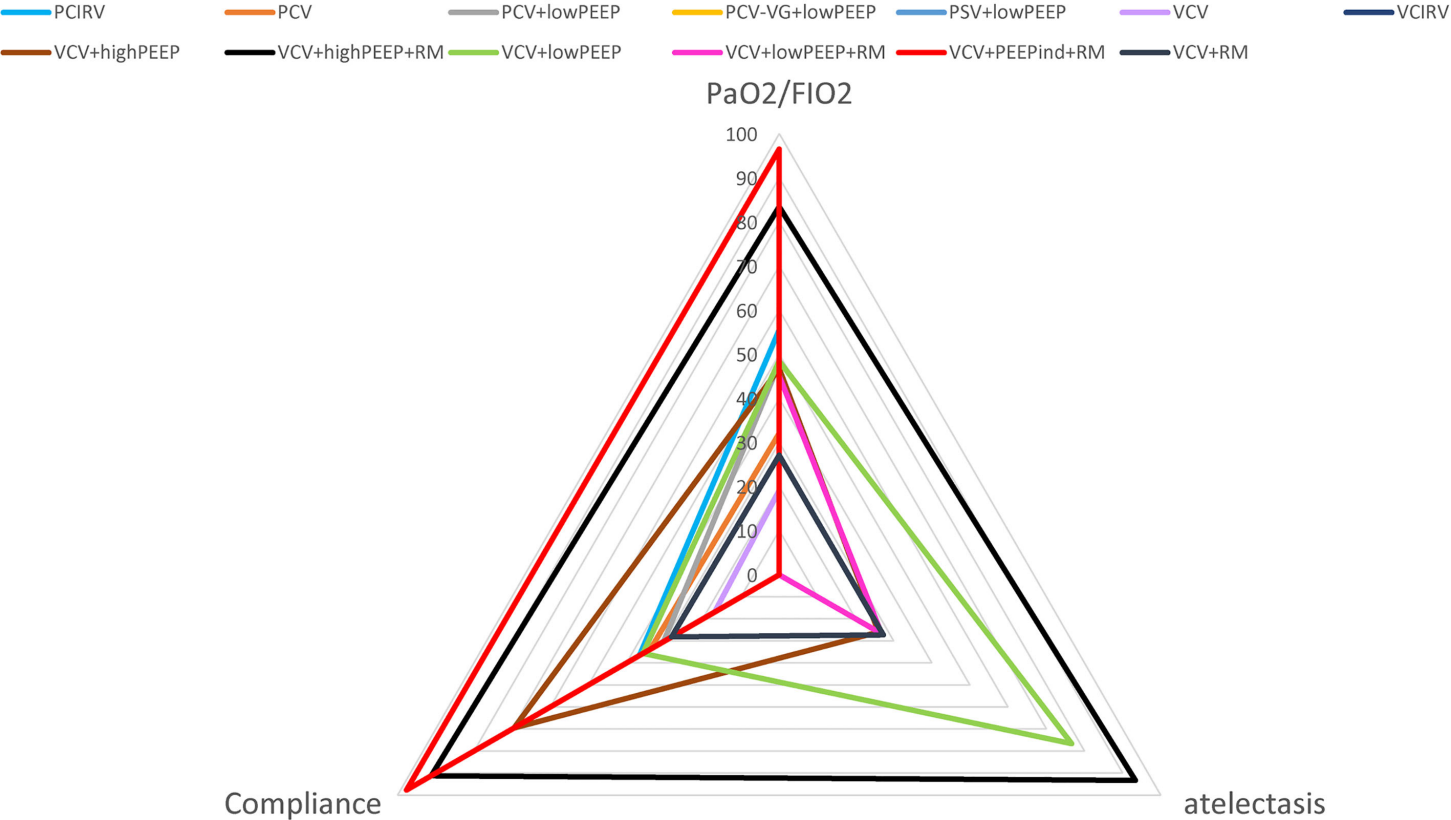
SUCRA ranking of the results in this meta-analysis.

The network meta-analysis revealed that the ventilation strategy VCV+PEEPind+RM was more effective than PCV, PCV+lowPEEP, pressure control - volume assurance ventilation with low positive expiratory end pressure (PCV-VG+lowPEEP), pressure-controlled inverse ratio ventilation (PCIRV), VCV, VCV+RM, VCV+lowPEEP or volume-controlled ventilation with high positive expiratory end pressure (VCV+highPEEP) in improving lung compliance, MD [95% CI] and [quality of evidence]: 29.47 [18.45, 40.48] [moderate], 29.85 [19.45, 40.24] [moderate], 24.18 [13.16, 35.20] [low], 29.24 [14.52, 43.96] [low], 32.45 [19.19, 45.71] [low], 30.51 [19.48, 41.54] [moderate], 28.98 [20.24, 37.72] [low], 19.83 [10.45, 29.22] [low]. VCV+PEEPind+RM is the best method to improve lung compliance (SUCRA: 0.977) (**Figure 5**). The supporting information shows network plots of intraoperative lung compliance for various ventilation strategies, MD values and the 95% CIs and credibility (**Figures S3, S6**) and SUCRA rankings (**Figure 6**).

#### 3.2.1 Risk of literature bias, article quality assurance, heterogeneity, consistency and sensitivity

The risk of bias assessment for different ventilation strategies is shown in the supporting information (**Figures S7, S8**). In summary, 1 study (42) was assessed as having a high bias risk and was excluded. Two studies were assessed as being unclear (33, 35), and the rest deemed to be low risk (12, 20–32, 34, 36–41). All studies were symmetrically distributed within the funnel plot, indicating no publication bias (supporting information **Figures S9–S11**). Overall heterogeneity for each measure is as follows: PaO_2_/FiO_2_ *I^2^* = 45.4%, pulmonary atelectasis test *I^2^* = 14%, lung compliance *I^2^* = 31.2% (supporting information **Figures S12–S14**). When excluding each study from the analysis individually, we found the quality of the article by Tuncali et al. (42).The *P*-values of the consistency tests were all > 0.05, and no inconsistency was found between the direct and indirect evidence (supporting information **Figures S15–S17**). For network meta-analysis estimates, the quality of evidence assessed by CINeMA analysis ranged from very low to high (supporting information **Tables S1–S3**).

## 4. Discussion

According to the network meta-analysis results, we found that the ventilation strategies VCV+PEEPind+RM were superior to 10 ventilation strategies in improving intraoperative PaO_2_/FiO_2_ and lung compliance. Of the 5 ventilation strategies for postoperative atelectasis, VCV+highPEEP+RM was shown to be the most effective in reducing atelectasis elicited by inflammation.

When compared with a fixed PEEP, individualized maneuvers on PEEP were found to offset end-expiratory volume, improve respiratory mechanics (44), reduce intrapulmonary shunt, permit a greater intraoperative respiratory compliance, enhance oxygenation ability, and improve intraoperative ventilation of patients, but these effects were not maintained early postoperatively (41, 45). It has been reported that an individualized PEEP core decreased postoperative pulmonary atelectasis while improving intraoperative oxygenation and driving pressures and minimizing complications (46). Taken together the latter results are in good agreement with our present findings, namely that ventilation strategies involving individualized patient PEEP are much more effective compared to other outcome evaluations.

The ventilation strategies VCV+PEEPind+RM were associated with optimal lung compliance. This is probably because PEEP maintains elastic retraction and enhances lung compliance, keeps alveoli open at the end of respiration, increases the functional residual air volume, causes alveoli to expand in a high applicable residual air volume state, and avoids excessive lung expansion and contraction during inspiration and expiration, thus reducing alveolar destruction (47).

Biological trauma caused by mechanical ventilation includes excessive alveolar expansion, periodic pulmonary atelectasis, immune cell activation and spillover of inflammatory mediators into the blood circulation. Several clinical studies have reported elevated concentrations of pro-inflammatory cytokines, such as interleukins 1, 6 and 8 and TNF-α in atelectasis, all of which are associated with inflammatory injury (48–50). Protective ventilation at a lower tidal volume and a higher PEEP level may reduce the negative cumulative effects of mechanical ventilation when the systemic inflammatory response syndrome occurs. Alveolar recruitment facilitates lung function and gas exchange, but protective mechanical ventilation strategies will likely reduce the generation of both local and systemic mediators of inflammation. Although we know that local immune disorders are closely associated with postoperative pulmonary atelectasis, clinical statistics are lacking, with only 1 of 23 included papers NMA describing perioperative TNF-α alterations, and noting that PCIRV may reduce the release of TNF-α and may prevent VCV-induced lung injury (33). The benefits of RM will be most marked when incorporated into a regimen of protective intrapulmonary ventilation (51, 52). Compared to zero PEEP or PEEP alone, pulmonary RM have been demonstrated to increase end-expiratory lung volume, improve compliance and reduce chest wall elasticity during laparoscopic procedures (53). However, multiple studies have failed to demonstrate that temporary improvements in lung mechanics or oxygenation are extended to the postoperative setting (12, 30).

Although the results of the present study have shown that VCV+highPEEP+RM has the greatest potential to reduce postoperative atelectasis, no statistically significant differences were found in the incidence of postoperative atelectasis between VCV+highPEEP+RM and VCV+lowPEEP. This result may be well be due to the relatively short operation times (average 2.5 h) for the 2 ventilation methods included in the analysis, perhaps being insufficient to have influenced the incidence of postoperative atelectasis. A more meaningful comparison would be the ventilation modes of VCV+highPEEP+RM and VCV+lowPEEP for longer surgery time (> 5 h).

This study had several limitations. First, it did not include every possible ventilation strategy. For example, the ventilation strategy volume-controlled equal ratio ventilation with high positive PEEP and RM was not studied. Second, different RMs affect outcome indicators, yet we did not differentiate between RMs. It would have been difficult to do so because the number of RCTs distinguishing between RMs is minimal. Third, tidal volume was not determined, and the effect of tidal volume on lung function was not explored. This is mainly because all tests adopted a protective ventilation strategy. Ventilation strategy metrics are available according to SUCRA, but there was no statistical difference between the best and next-best ranked strategy. Finally, intraoperative pulmonary diffusion function was not assessed because of the lack of data on forced expiratory volume in the first second and forced vital capacity.

## 5. Conclusions

VCV+PEEPind+RM is the optimal ventilation strategy for patients with obesity in increasing intraoperation PaO_2_/FiO_2_ and lung compliance, and among the five ventilation strategies for postoperative atelectasis, VCV+highPEEP+RM had the greatest potential to reduce atelectasis caused by inflammation.

## Data availability statement

The original contributions presented in the study are included in the article/**Supplementary Material**. Further inquiries can be directed to the corresponding author.

## Author contributions

CY conceived and designed the study. JW and JZ selected the articles and extracted the data. XH and GD analyzed the data. JW wrote the first draft of the manuscript. CZ and WZ independently assessed the risk of bias in the included studies. All authors interpreted the data and contributed to writing the final version of the manuscript. All authors agreed with the results and conclusions of this article. The corresponding author attests that all listed authors meet authorship criteria and that no others meeting the criteria have been omitted. All authors contributed to the article and approved the submitted version.

## Funding

This trial was supported by the Intelligent Medicine Project of Chongqing Medical University, China (Grant No: ZHYX202116), and CSA Clinical Research Fund (CSA-A2021-05).

## Conflict of interest

The authors declare that the research was conducted in the absence of any commercial or financial relationships that could be construed as a potential conflict of interest.

## Publisher’s note

All claims expressed in this article are solely those of the authors and do not necessarily represent those of their affiliated organizations, or those of the publisher, the editors and the reviewers. Any product that may be evaluated in this article, or claim that may be made by its manufacturer, is not guaranteed or endorsed by the publisher.
