# Supplementary material for: Optimized ventilation strategy for surgery on patients with obesity from the perspective of lung protection: A network meta-analysis
Source: Front Immunol. 2022 Oct 18;13:1032783. doi: 10.3389/fimmu.2022.1032783 (PMC9623268; doi:10.3389/fimmu.2022.1032783)
Supplement: Supplementary file 1 [file DataSheet_1.pdf]

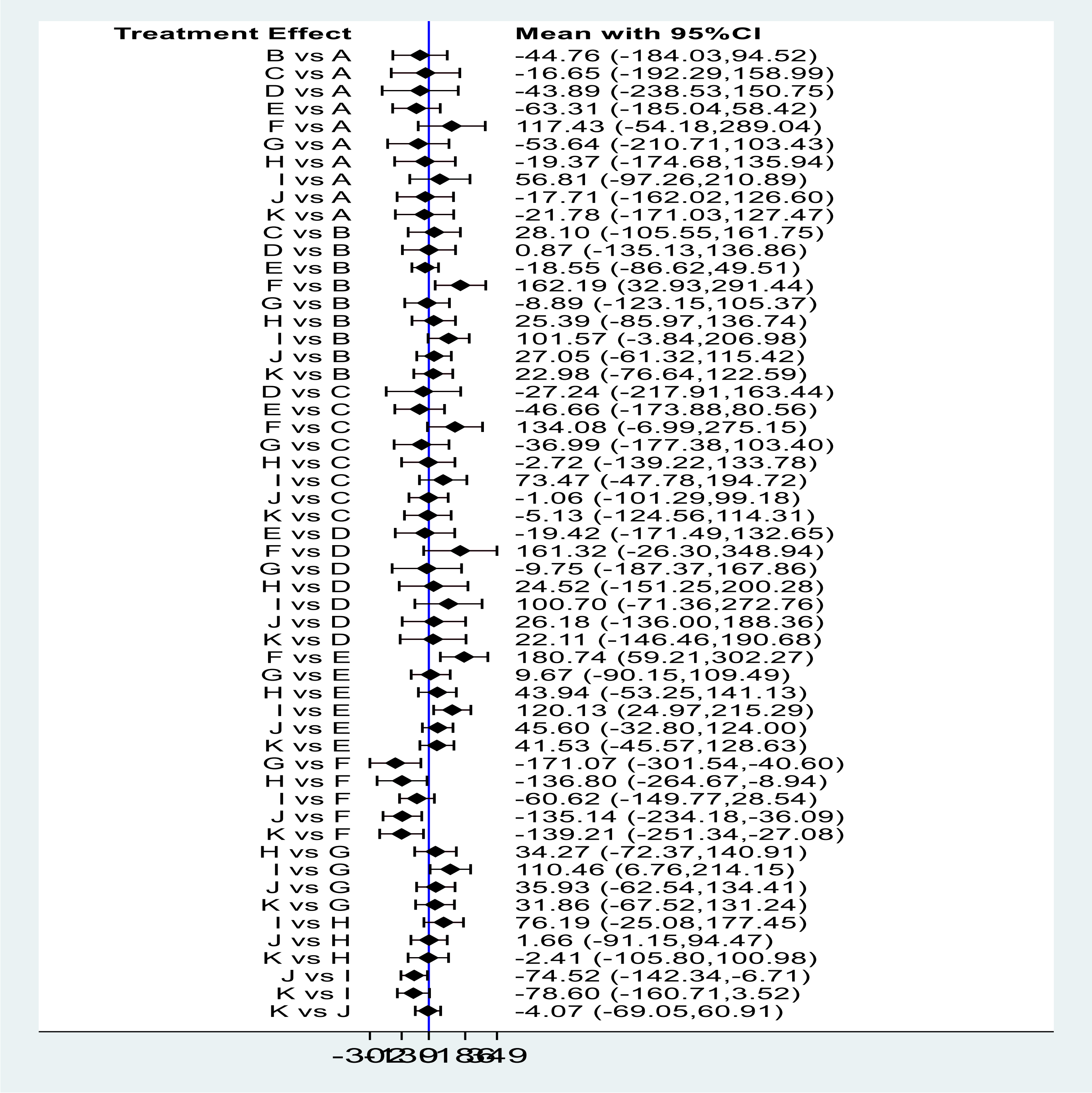

Abbreviation :

A = PCIRV

B = PCV

C = PCV+lowPEEP

D = PSV+lowPEEP

E = VCV

F = VCV+PEEPind+RM

G = VCV+RM

H = VCV+highPEEP

I = VCV+highPEEP+RM

J = VCV+lowPEEP

K = VCV+lowPEEP+RM

Figuer S1 forest plot of PaO_2/FiO_2 based on different ventilation strategies

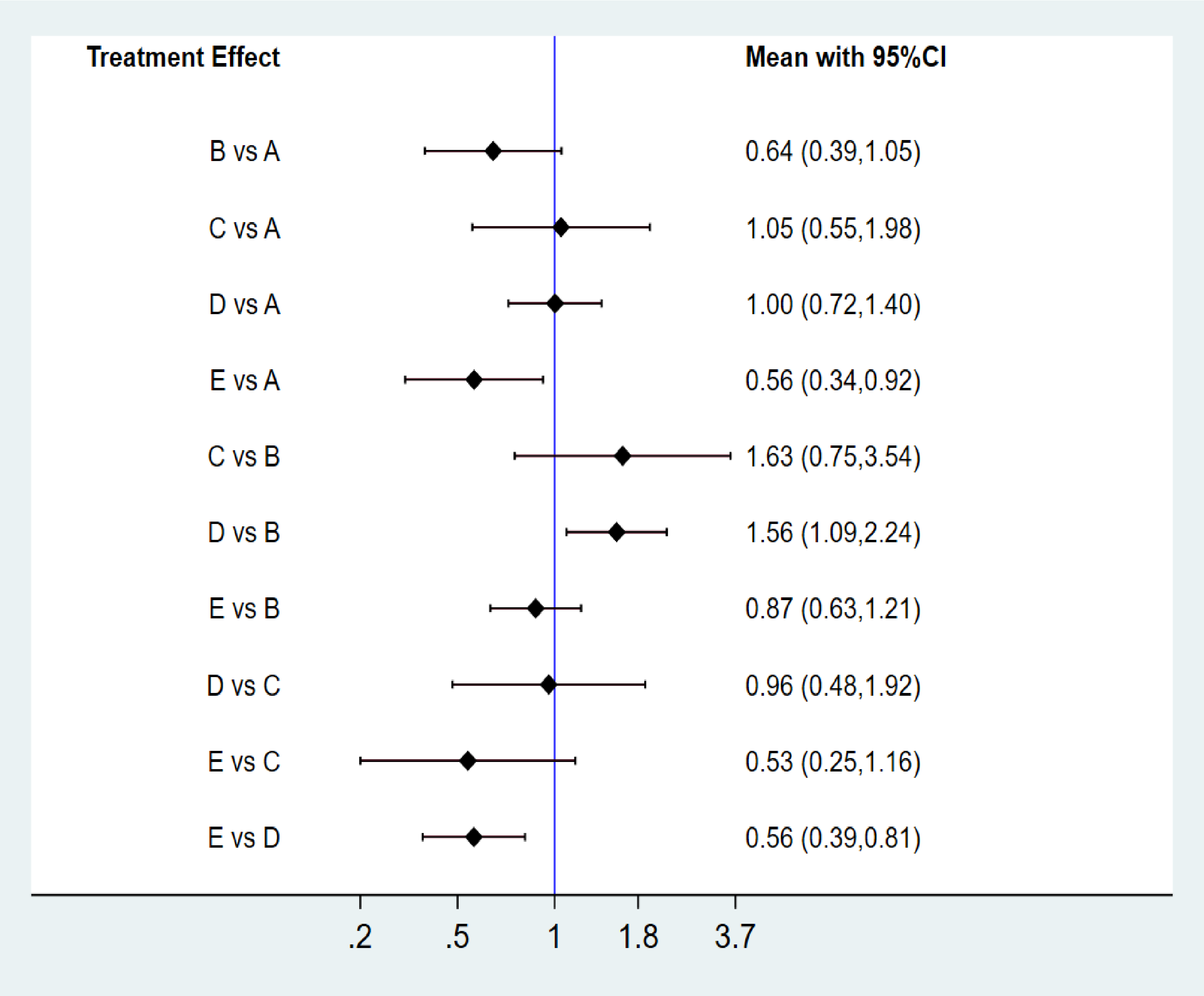

Abbreviation :
A = VCV+RM
B =VCV+lowPEEP
C=VCV+highPEEP
D =VCV+lowPEEP+RM
E = VCV+highPEEP+RM

Figure S2 forest plot of pulmonary atelectasis incidence rate based on different ventilation strategies

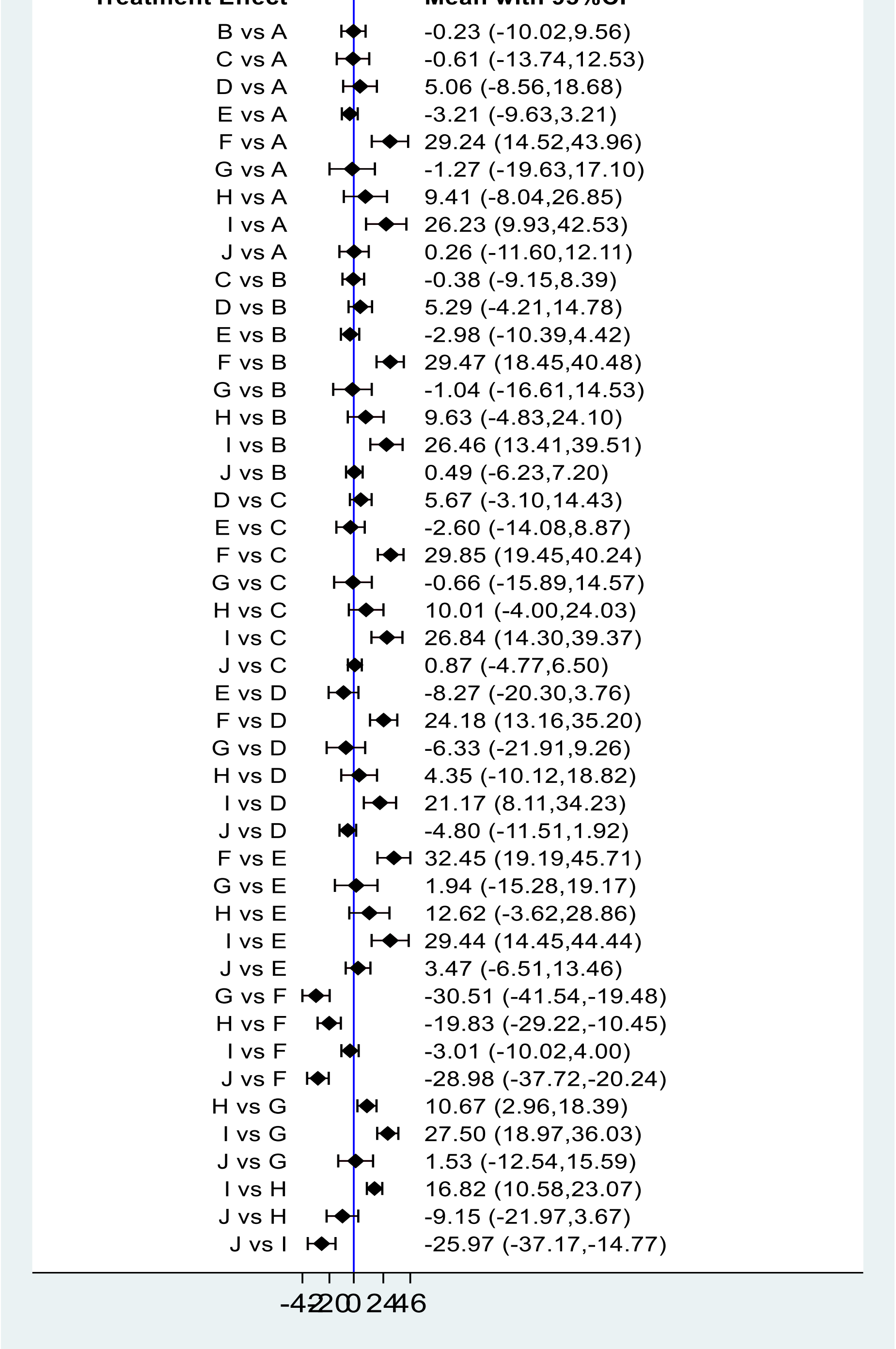

Abbreviation ：

A = PCIRV

B = PCV

C = PCV+lowPEEP

D = PCV-CG+lowPEEP

E = VCV

F = VCV+PEEPind+RM

G = VCV+RM

H = VCV+highPEEP

I = VCV+highPEEP+RM

J = VCV+lowPEEP

Figure S3 forest plot of pulmonary compliance based on different ventilation strategies

Treatment	SUCRA	PrBest	MeanRank
PCIRV	55.5	6.7	5.5
PCV	32.1	0.1	7.8
PCV+lowPEEP	47.9	1.2	6.2
PSV+lowPEEP	48.1	7.6	6.2
VCV	19.0	0.0	9.1
VCV+PEEPind+RM	96.5	76.9	1.4
VCV+RM	27.1	0.1	8.3
VCV+highPEEP	46.8	0.6	6.3
VCV+highPEEP+RM	83.5	6.7	2.6
VCV+lowPEEP	48.5	0.0	6.1
VCV+lowPEEP+RM	45.1	0.1	6.5

Figure S4 treatment relative ranking of PaO2/FiO2

Treatment	SUCRA	PrBest	MeanRank
vcv+rm	27.3	0.4	3.9
vcv+lowpeep	76.6	19.3	1.9
vcv+highpeep	26.0	4.5	4.0
vcv+lowpeep+rm	26.8	0.0	3.9
vcv+highpeep+rm	93.3	75.7	1.3

Figure S5 treatment relative ranking of pulmonary atelectasis

Treatment	SUCRA	PrBest	MeanRank
PCIRV	36.6	0.0	6.7
PCV	33.8	0.0	7.0
PCV+lowPEEP	30.3	0.0	7.3
PCV-CG+lowPEEP	60.3	0.0	4.6
VCV	16.9	0.0	8.5
VCV+PEEPind+RM	97.7	79.1	1.2
VCV+RM	28.2	0.0	7.5
VCV+highPEEP	69.5	0.0	3.7
VCV+highPEEP+RM	91.2	20.9	1.8
VCV+lowPEEP	35.7	0.0	6.8

Figure S6 treatment relative ranking of pulmonary compliance

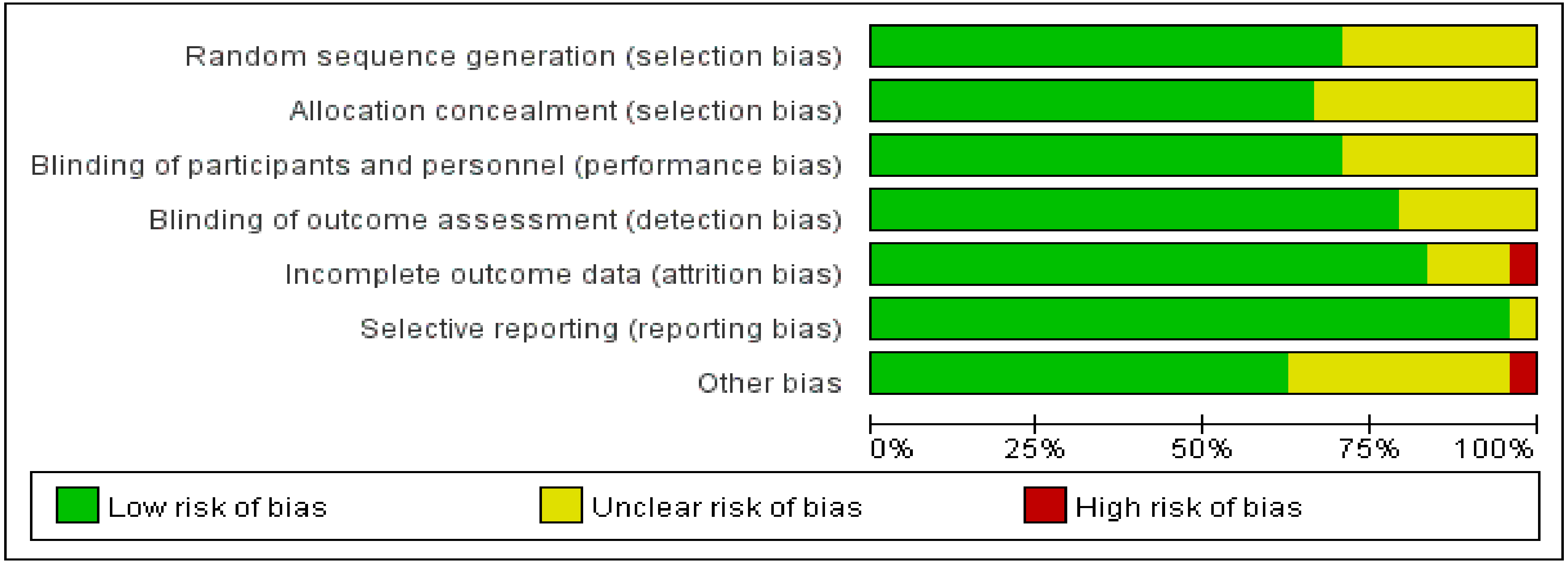

Figure S7 Risk map for bias assessment of different ventilation strategies

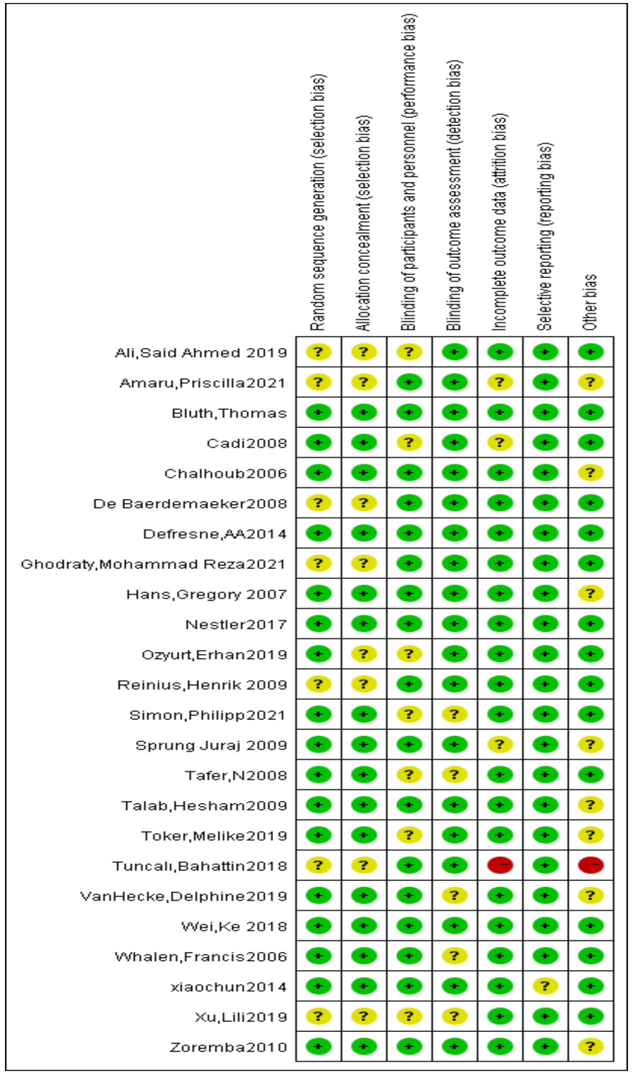

Figure S8 Risk map for bias assessment of different ventilation strategies

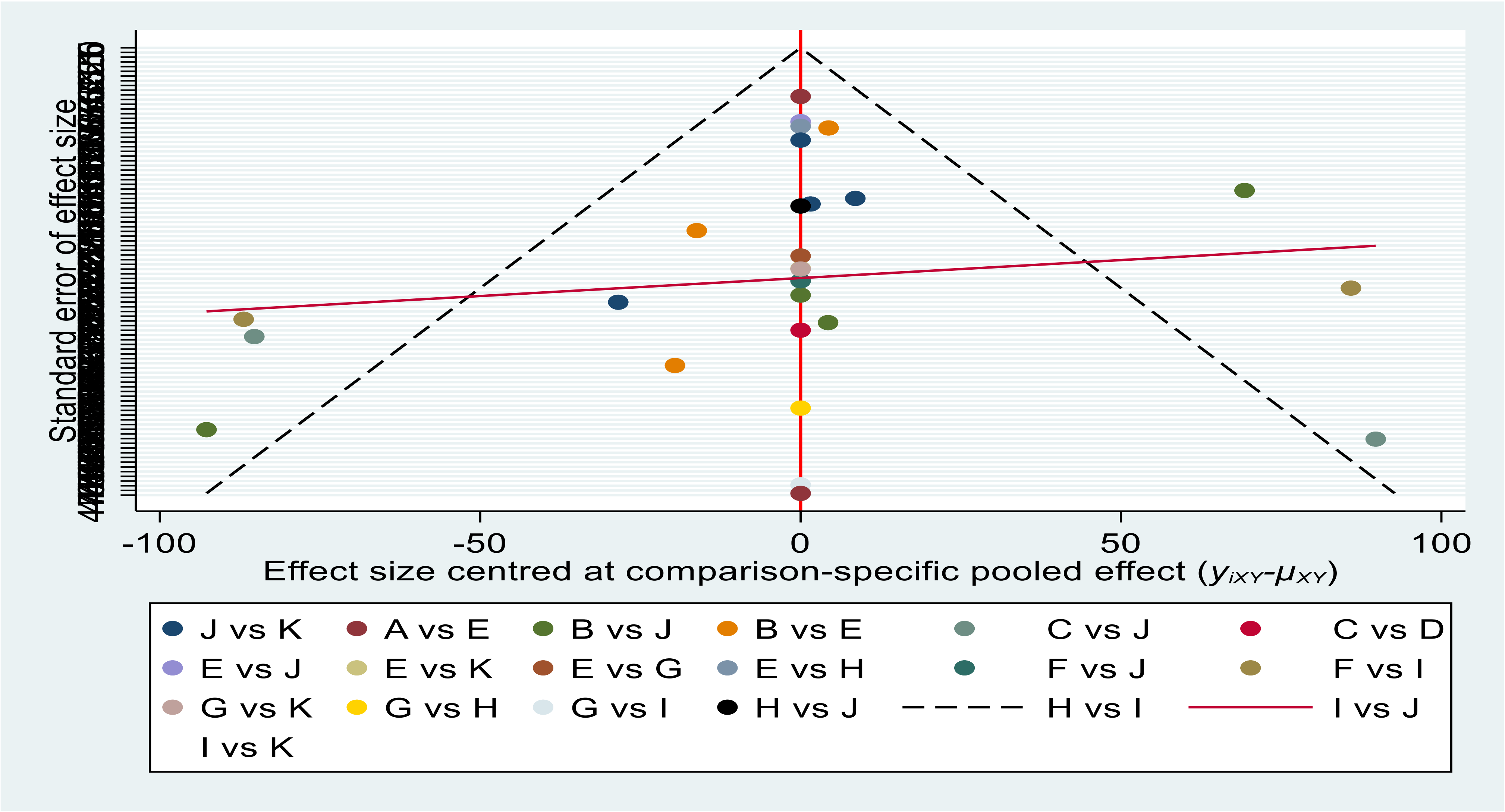

Figure S9 Funnel plot of intraoperative PaO2/FiO2

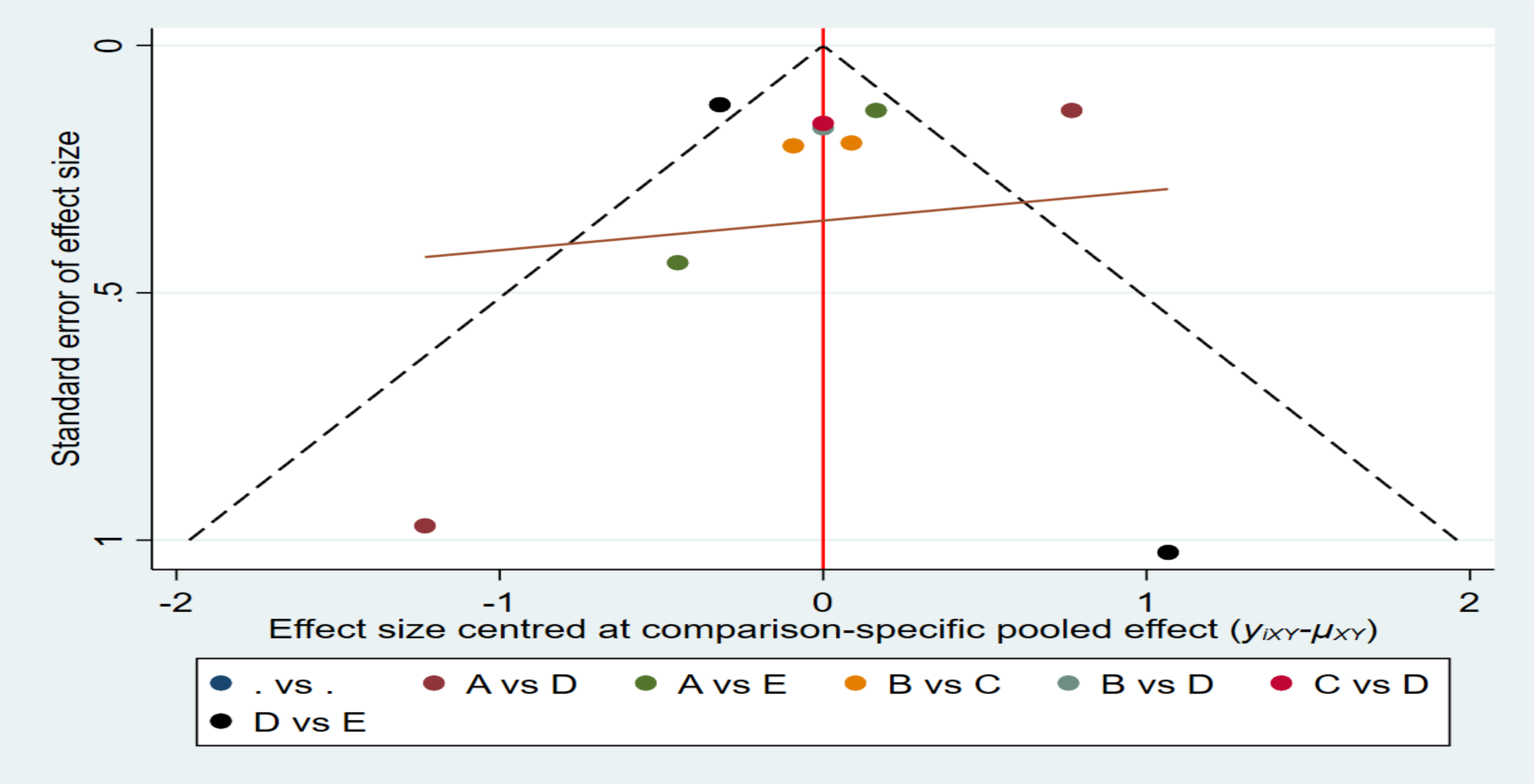

Figure S10 Funnel plot of intraoperative pulmonary atelectasis

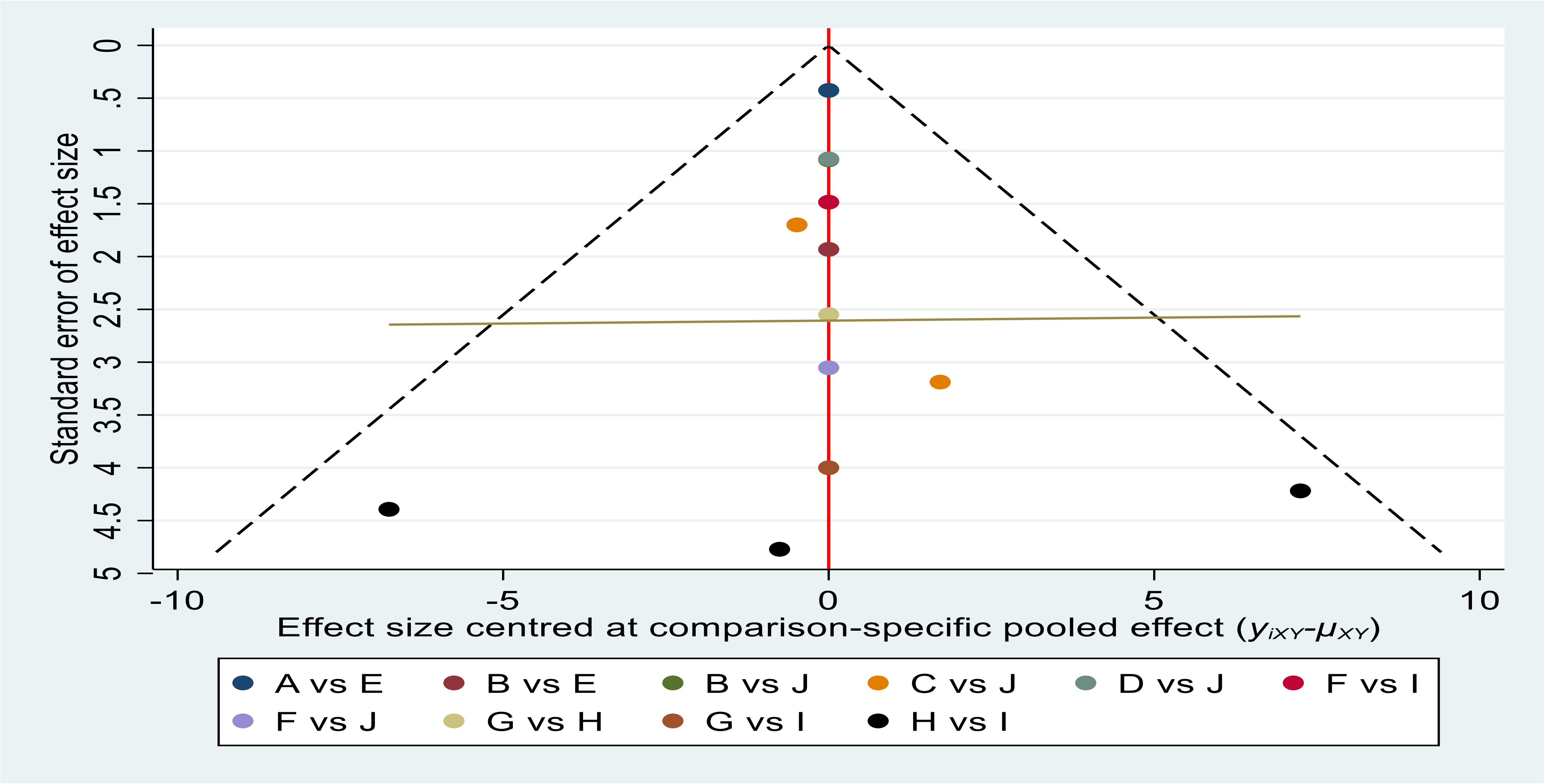

Figure S11 Funnel plot of intraoperative lung compliance

Side	Direct Coef.	Std. Err.	Indirect Coef.	Std. Err.	Difference Coef.	Std. Err.	P>|z|	tau
A E *	-64	62.89547	-32.13456	422.4147	-31.86544	427.0714	0.941	62.68285
B D *	.8600159	69.39157	89.54541	7553.642	-88.68539	7554.021	0.991	62.57849
B E	1.627078	37.27709	-111.1997	80.09105	112.8268	88.33588	0.202	60.70188
B J	-35.99979	66.14547	76.77834	58.68553	-112.7781	88.42635	0.202	60.70879
C J *	-1.051394	51.14689	-30.87156	4277.252	29.82017	4277.544	0.994	62.58218
E G	46.997	67.32887	-43.78492	80.41006	90.78193	104.8731	0.387	63.59527
E H	62.59723	65.8274	13.92821	82.73728	48.66903	105.6847	0.645	65.29846
E J	58.65141	66.4733	37.01748	54.18429	21.63393	85.76887	0.801	66.00905
E K	59.25208	69.76659	27.44558	62.24851	31.8065	93.5038	0.734	65.71644
F I *	-56.0511	50.0428	-98.14022	149.0087	42.08912	157.9821	0.790	65.33138
F J	-188.2114	64.43882	-65.44781	73.61531	-122.7636	97.87039	0.210	59.49859
G H	46.50695	76.07331	19.11121	85.00186	27.39575	114.0836	0.810	65.75725
G I	175.6266	76.82364	54.17679	70.93168	121.4498	104.9456	0.247	61.37201
G K	11.74059	69.41714	59.56494	80.58868	-47.82435	106.4239	0.653	65.32391
H I	128.164	78.9101	36.48874	69.22606	91.67522	105.1936	0.383	63.24478
H J	-3.979085	66.70006	9.301344	74.15001	-13.28043	99.72285	0.894	65.97587
I J	-46.72584	38.27251	-156.02	66.57129	109.2942	76.83021	0.155	59.40844
I K	4.495489	58.63821	-136.8049	50.22941	141.3004	77.21297	0.067	56.18022
J K	-24.08977	37.50568	60.032	67.04797	-84.12177	76.77739	0.273	61.72991

Figure S12 Differences in direct and indirect comparisons related to PaO2/FiO2
Abbreviation ：A = PCIRV,B = PCV, C = PCV+lowPEEP, D = PSV+lowPEEP, E = VCV, F = VCV+PEEPind+RM, G = VCV+RM, H = VCV+highPEEP, I = VCV+highPEEP+RM, J = VCV+lowPEEP, K = VCV+lowPEEP+RM

Side	Direct		Indirect		Difference			tau
	Coef.	Std. Err.	Coef.	Std. Err.	Coef.	Std. Err.	P>|z|	
A C *	.0452795	.3261097	.6679511	57.51022	-.6226716	57.50957	0.991	.1242545
A D *	.0019929	.1712385	.3133383	28.73329	-.3113454	28.73379	0.991	.1242548
B D *	.4886426	.5945762	.1291225	1.602204	.3595201	1.716788	0.834	.5733595
B E	.	.	.	.	.	.	.	.
C D *	-.0432867	.3543845	.5793854	57.53398	-.6226721	57.53373	0.991	.1242546
D E *	-.5343369	.5969788	-.8920711	1.599194	.3577342	1.716542	0.835	.5732662

Figure S13 Differences in direct and indirect comparisons related topulmonary atelectasis

Abbreviation :
A = VCV+RM, B= VCV+lowPEEP, C = VCV+highPEEP, D= VCV+lowPEEP+RM, E= VCV+highPEEP+RM

Side	Direct Coef.	Std. Err.	Indirect Coef.	Std. Err.	Difference Coef.	Std. Err.	P>|z|	tau
A F *	-3.199999	3.307459	-9.070836	66.00112	5.870837	66.08394	0.929	3.279881
B F *	-2.999988	3.806993	3.290756	67.92395	-6.290744	68.03047	0.926	3.280387
B K *	.5	3.453905	-7.598201	76.68835	8.098201	76.76609	0.916	3.279247
C K *	.8666755	2.881098	.467553	266.5439	.3991225	266.5618	0.999	3.260003
D K *	-4.800001	3.430187	.5065121	335.4848	-5.306513	335.5012	0.987	3.257328
E J *	-5.900002	3.862232	52.51481	684.4802	-58.41481	684.4888	0.932	3.253171
G J *	-3.000097	3.585325	-24.58409	154.7564	21.58399	154.7984	0.889	3.263366
G K *	-28.99992	4.469846	-7.820568	131.4602	-21.17935	131.5374	0.872	3.264898
H I *	13	2.54951	-7.974988	10.20021	20.97499	10.82332	0.053	1.78e-09
H J *	23	4	43.9849	8.691563	-20.9849	10.82327	0.053	1.03e-06
I J *	16.82141	3.187864	44.38431	373.6199	-27.5629	373.6416	0.941	3.258452

Figure S14 Differences in direct and indirect comparisons related to lung compliance
Abbreviation： A = PCIRV, B = PCV , C = PCV+lowPEEP, D = PCV-CG+lowPEEP, E = VCV
F = VCV+PEEPind+RM, G = VCV+RM, H = VCV+highPEEP, I = VCV+highPEEP+RM, J = VCV+lowPEEP

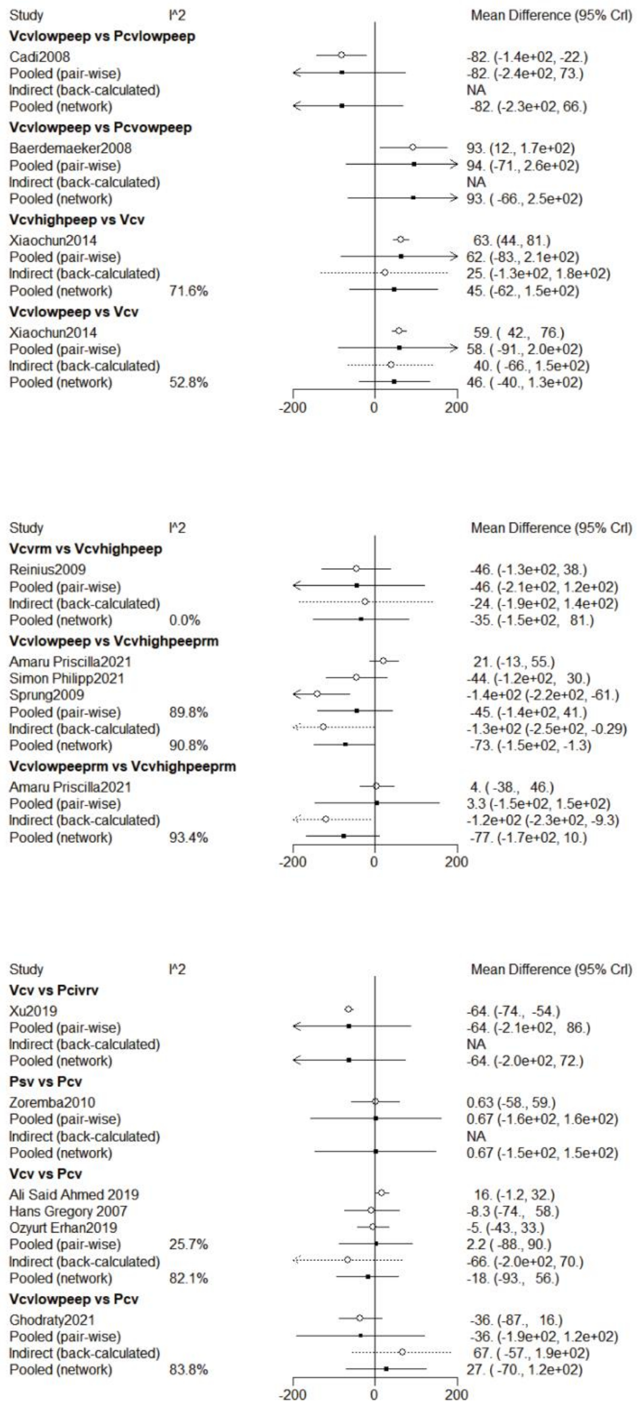

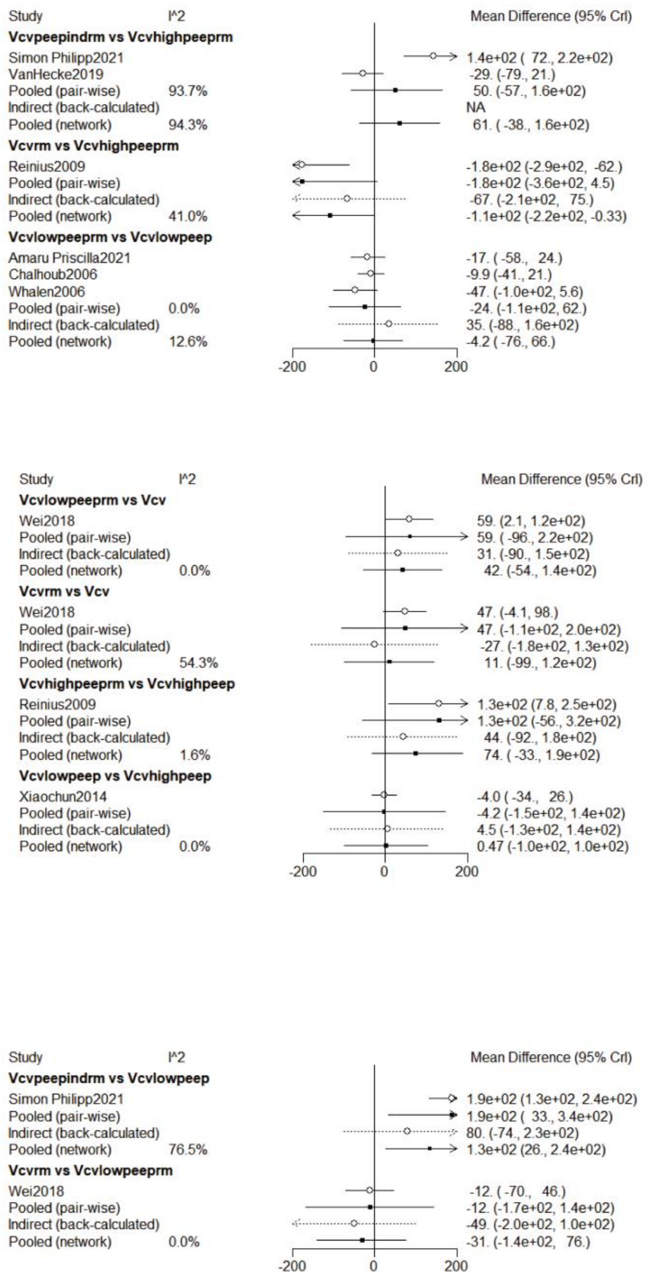

Figure S15 forest plot of heterogeneity in PaO2/FiO2

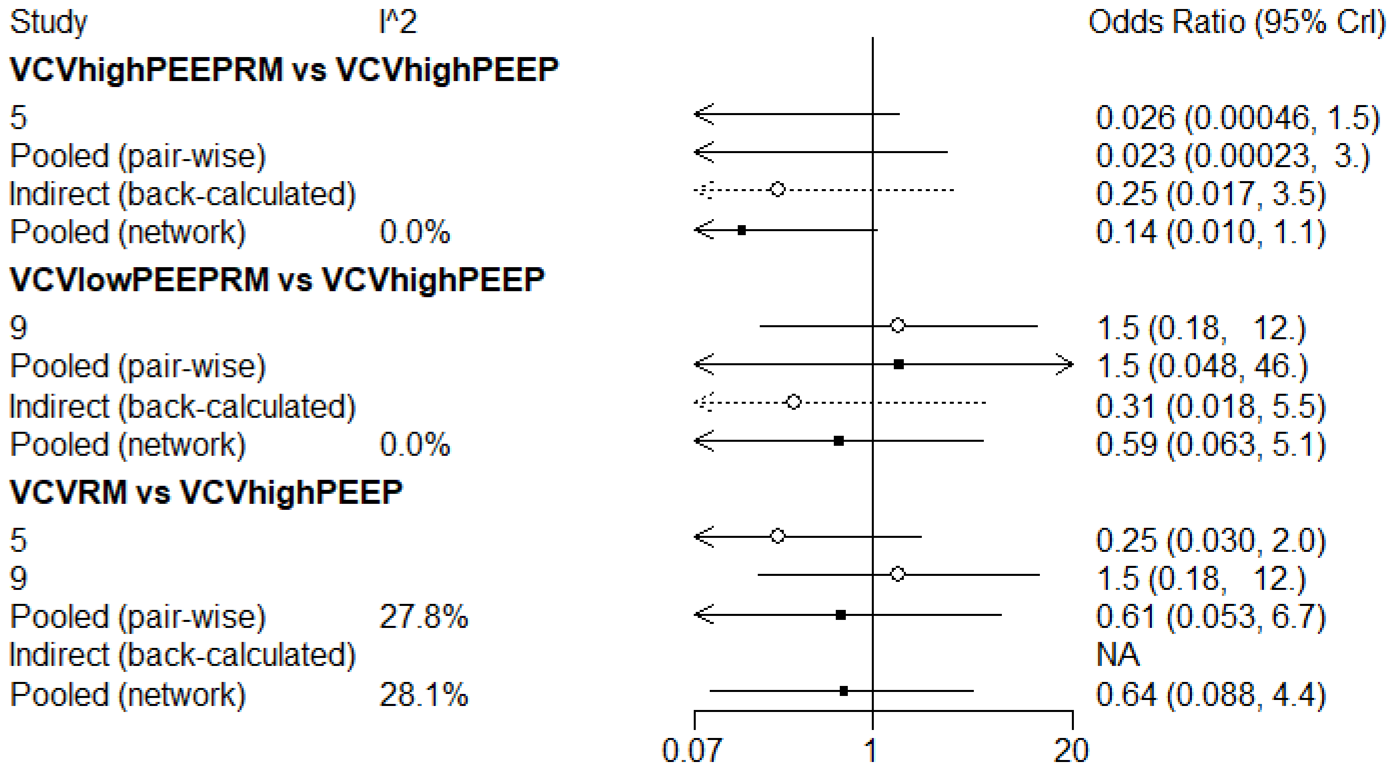

Figure S16 forest plot of heterogeneity in pulmonary atelectasis

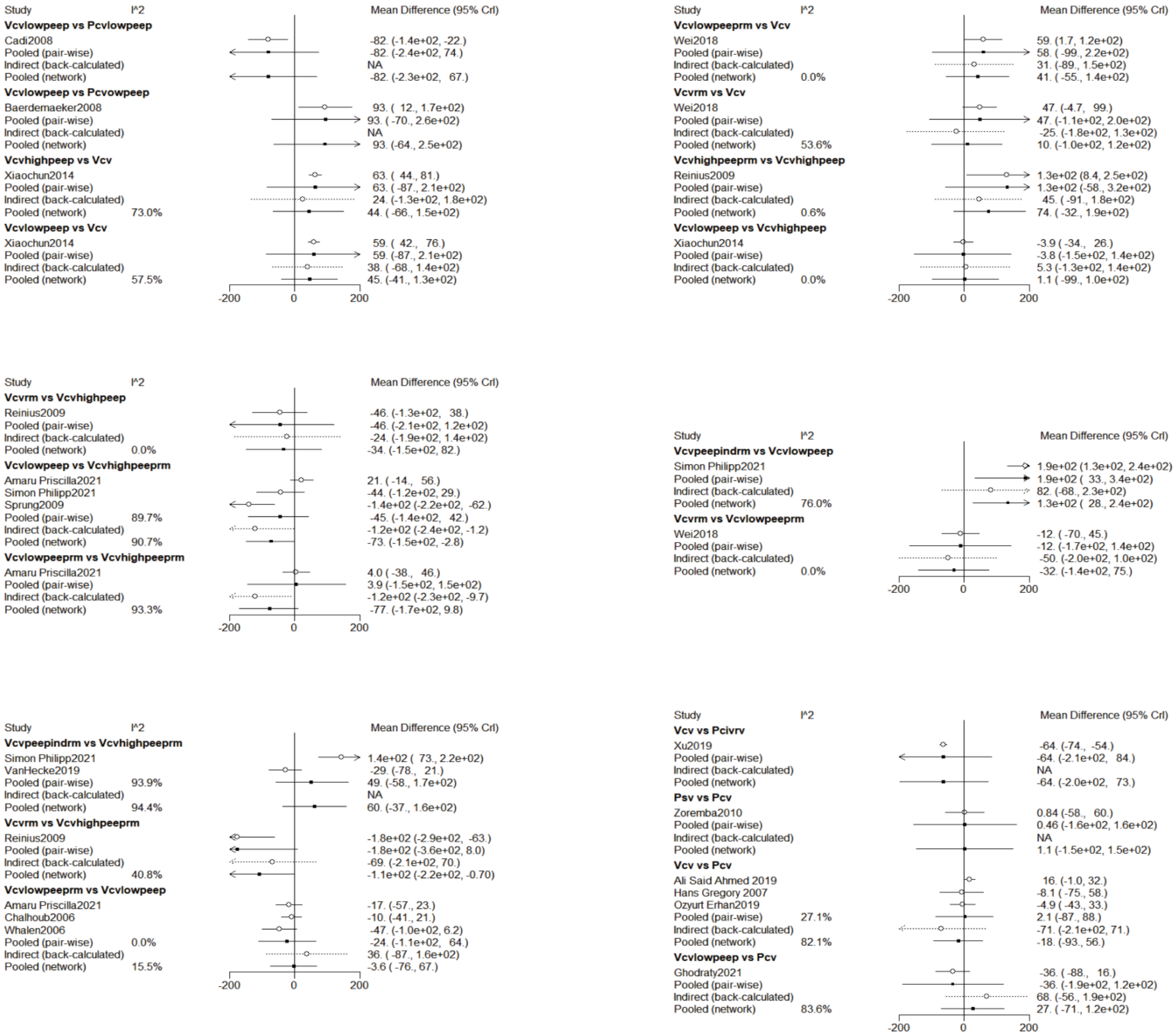

Figure S17 forest plot of heterogeneity in lung compliance

PCIRV:VCV	1	Some concerns	Low risk	No concerns	Major concerns	No concerns	Major concerns	Very low
PCV:PSV	1	No concerns	Low risk	No concerns	Major concerns	No concerns	Major concerns	Very low
PCV:VCV	3	No concerns	Low risk	No concerns	Major concerns	No concerns	No concerns	Low
PCV:VCV+lowPEEP	1	No concerns	Low risk	No concerns	Major concerns	No concerns	No concerns	Low
PCV+lowPEEP:VCV+lowPEEP	2	No concerns	Low risk	No concerns	Major concerns	No concerns	Major concerns	Very low
VCV:VCV+RM	1	No concerns	Low risk	No concerns	Major concerns	No concerns	No concerns	Low
VCV:VCV+highPEEP	1	No concerns	Low risk	No concerns	Major concerns	No concerns	No concerns	Low
VCV:VCV+lowPEEP	1	No concerns	Low risk	No concerns	Major concerns	No concerns	No concerns	Low
VCV:VCV+lowPEEP+RM	1	No concerns	Low risk	No concerns	Major concerns	No concerns	No concerns	Low
VCV+highPEEP+RM:VCV+PEEPind+RM	2	No concerns	Low risk	No concerns	Major concerns	No concerns	No concerns	Low
VCV+lowPEEP:VCV+PEEPind+RM	1	No concerns	Low risk	No concerns	No concerns	Major concerns	No concerns	Low
VCV+highPEEP:VCV+RM	1	No concerns	Low risk	No concerns	Major concerns	No concerns	No concerns	Low
VCV+highPEEP+RM:VCV+RM	1	No concerns	Low risk	No concerns	No concerns	Major concerns	No concerns	Low
VCV+lowPEEP+RM:VCV+RM	1	No concerns	Low risk	No concerns	Major concerns	No concerns	No concerns	Low
VCV+highPEEP:VCV+highPEEP+RM	1	No concerns	Low risk	No concerns	Major concerns	No concerns	No concerns	Low
VCV+highPEEP:VCV+lowPEEP	1	No concerns	Low risk	No concerns	Major concerns	No concerns	No concerns	Low
VCV+highPEEP+RM:VCV+lowPEEP	3	No concerns	Low risk	No concerns	No concerns	Major concerns	No concerns	Low
VCV+highPEEP+RM:VCV+lowPEEP+RM	1	No concerns	Low risk	No concerns	No concerns	Major concerns	Major concerns	Very low
VCV+lowPEEP:VCV+lowPEEP+RM	3	No concerns	Low risk	No concerns	Major concerns	No concerns	No concerns	Low
PCIRV:PCV	0	No concerns	Low risk	No concerns	Major concerns	No concerns	Major concerns	Very low
PCIRV:PCV+lowPEEP	0	No concerns	Low risk	No concerns	Major concerns	No concerns	Major concerns	Very low
PCIRV:PSV	0	No concerns	Low risk	No concerns	Major concerns	No concerns	Major concerns	Very low
PCIRV:VCV+PEEPind+RM	0	No concerns	Low risk	No concerns	Major concerns	No concerns	Major concerns	Very low
PCIRV:VCV+RM	0	No concerns	Low risk	No concerns	Major concerns	No concerns	Major concerns	Very low
PCIRV:VCV+highPEEP	0	No concerns	Low risk	No concerns	Major concerns	No concerns	Major concerns	Very low
PCIRV:VCV+highPEEP+RM	0	No concerns	Low risk	No concerns	Major concerns	No concerns	Major concerns	Very low
PCIRV:VCV+lowPEEP	0	No concerns	Low risk	No concerns	Major concerns	No concerns	Major concerns	Very low
PCIRV:VCV+lowPEEP+RM	0	No concerns	Low risk	No concerns	Major concerns	No concerns	Major concerns	Very low
PCV:PCV+lowPEEP	0	No concerns	Low risk	No concerns	Major concerns	No concerns	Major concerns	Very low
PCV:VCV+PEEPind+RM	0	No concerns	Low risk	No concerns	No concerns	Major concerns	Major concerns	Very low
PCV:VCV+RM	0	No concerns	Low risk	No concerns	Major concerns	No concerns	Major concerns	Very low
PCV:VCV+highPEEP	0	No concerns	Low risk	No concerns	Major concerns	No concerns	Major concerns	Very low
PCV:VCV+highPEEP+RM	0	No concerns	Low risk	No concerns	No concerns	Major concerns	Major concerns	Very low

Table S1 CINeMA table of PaO2/FIO2 based on different ventilation strategies

Comparison	Number of studies	Within-study bias	Reporting bias	Indirectness	Imprecision	Heterogeneity	Incoherence	Confidence rating
2:04	1	No concerns	Low risk	No concerns	Major concerns	No concerns	No concerns	Moderate
2:06	1	No concerns	Low risk	No concerns	Major concerns	No concerns	No concerns	Moderate
3:05	1	Some concerns	Low risk	No concerns	No concerns	Major concerns	No concerns	Moderate
3:06	2	No concerns	Low risk	No concerns	Major concerns	No concerns	No concerns	Moderate
4:05	1	No concerns	Low risk	No concerns	Major concerns	No concerns	No concerns	Moderate
4:06	1	No concerns	Low risk	No concerns	No concerns	Major concerns	No concerns	Moderate
5:06	1	Some concerns	Low risk	No concerns	No concerns	Major concerns	No concerns	Moderate
2:03	0	No concerns	Low risk	No concerns	Major concerns	No concerns	No concerns	Moderate
2:05	0	No concerns	Low risk	No concerns	Major concerns	No concerns	No concerns	Moderate
3:04	0	No concerns	Low risk	No concerns	No concerns	Major concerns	No concerns	Moderate

Table S2 CINeMA table pulmonary atelectasis based on different ventilation strategies

PCIRV:VCV+highPEEP+RM	No concerns	Low risk	No concerns	No concerns	Major concerns	Some conc	Low
PCIRV:VCV+lowPEEP	No concerns	Low risk	No concerns	Major concerns	No concerns	Some conc	Low
PCV:PCV+lowPEEP	No concerns	Low risk	No concerns	Major concerns	No concerns	Some conc	Low
PCV:PCV-CG+lowPEEP	No concerns	Low risk	No concerns	Major concerns	No concerns	Some conc	Low
PCV:VCV+PEEPind+RM	No concerns	Low risk	No concerns	No concerns	No concerns	Some conc	Moderat
PCV:VCV+RM	No concerns	Low risk	No concerns	Major concerns	No concerns	Some conc	Low
PCV:VCV+highPEEP	No concerns	Low risk	No concerns	Major concerns	No concerns	Some conc	Low
PCV:VCV+highPEEP+RM	No concerns	Low risk	No concerns	No concerns	Major concerns	Some conc	Low
PCV-CG+lowPEEP:PCV+lowPEEP	No concerns	Low risk	No concerns	Major concerns	No concerns	Some conc	Low
PCV+lowPEEP:VCV	No concerns	Low risk	No concerns	Major concerns	No concerns	Some conc	Low
PCV+lowPEEP:VCV+PEEPind+RM	No concerns	Low risk	No concerns	No concerns	No concerns	Some conc	Moderat
PCV+lowPEEP:VCV+RM	No concerns	Low risk	No concerns	Major concerns	No concerns	Some conc	Low
PCV+lowPEEP:VCV+highPEEP	No concerns	Low risk	No concerns	Major concerns	No concerns	Some conc	Low
PCV+lowPEEP:VCV+highPEEP+RM	No concerns	Low risk	No concerns	No concerns	Major concerns	Some conc	Low
PCV-CG+lowPEEP:VCV	No concerns	Low risk	No concerns	Major concerns	No concerns	Some conc	Low
PCV-CG+lowPEEP:VCV+PEEPind+RM	No concerns	Low risk	No concerns	No concerns	Major concerns	Some conc	Low
PCV-CG+lowPEEP:VCV+RM	No concerns	Low risk	No concerns	Major concerns	No concerns	Some conc	Low
PCV-CG+lowPEEP:VCV+highPEEP	No concerns	Low risk	No concerns	Major concerns	No concerns	Some conc	Low
PCV-CG+lowPEEP:VCV+highPEEP+RM	No concerns	Low risk	No concerns	No concerns	Major concerns	Some conc	Low
VCV:VCV+PEEPind+RM	No concerns	Low risk	No concerns	No concerns	No concerns	Some conc	Moderat
VCV:VCV+RM	No concerns	Low risk	No concerns	Major concerns	No concerns	Some conc	Low
VCV:VCV+highPEEP	No concerns	Low risk	No concerns	Major concerns	No concerns	Some conc	Low
VCV:VCV+highPEEP+RM	No concerns	Low risk	No concerns	No concerns	Major concerns	Some conc	Low
VCV:VCV+lowPEEP	No concerns	Low risk	No concerns	Major concerns	No concerns	Some conc	Low
VCV+PEEPind+RM:VCV+RM	No concerns	Low risk	No concerns	No concerns	No concerns	Some conc	Moderat
VCV+highPEEP:VCV+PEEPind+RM	No concerns	Low risk	No concerns	No concerns	Major concerns	Some conc	Low
VCV+lowPEEP:VCV+RM	No concerns	Low risk	No concerns	Major concerns	No concerns	Some conc	Low
VCV+highPEEP:VCV+lowPEEP	No concerns	Low risk	No concerns	Major concerns	No concerns	Some conc	Low
VCV+highPEEP+RM:VCV+lowPEEP	No concerns	Low risk	No concerns	No concerns	Major concerns	Some conc	Low

Table S3 CINeMA table of lung compliance based on different ventilation strategies
